# Evaluation of inter‐observer variability regarding aortic and mitral valve findings on transesophageal echocardiograms ordered for suspected endocarditis

**DOI:** 10.1111/echo.15400

**Published:** 2022-06-22

**Authors:** Kristina B. Moon, Matthew C. Tattersall, Maame Adoe, Fauzia Osman, Peter S. Rahko

**Affiliations:** ^1^ Department of Medicine University of Wisconsin School of Medicine and Public Health Madison Wisconsin USA

**Keywords:** infectious endocarditis, inter‐observer agreement, transesophageal echocardiogram, valvular mass, vegetation

## Abstract

**Background:**

Transesophageal echocardiography (TEE) is the gold standard for the detection of valvular vegetations (VV). Differentiating small VV from degenerative changes is challenging and prone to inter‐observer variability. We evaluated inter‐observer agreement regarding aortic (AV) and mitral valve (MV) findings on TEEs ordered for suspected infective endocarditis (IE).

**Methods:**

A total of 349 consecutive TEEs were evaluated. Studies were classified as “definite, possible, or no” IE with valve masses classified further by morphology. Nine faculty echocardiographers scored randomly selected TEEs of the AV (*N* = 38) and MV (*N* = 35). Inter‐reader variability was calculated using the Fleiss/Scott Kappa (Kf).

**Results:**

Positive blood cultures were present in 81% and 45% had definite IE by the modified Duke criteria. There was moderate reader agreement regarding the presence of a valvular mass for both the AV (*Kf* = .41, 95% CI [.30–.53]) and MV (*Kf* = .49, 95% CI [.34–.65]). For diagnosis of IE, there was fair agreement for the AV (*Kf *= .29, 95% CI [.18–.42]) and moderate agreement for the MV (*Kf *= .53, 95% CI [.36–.70]). Masses described as large, multi‐lobulated, or pedunculated were more frequently categorized as clinical IE, (*p* < .006, both valves), however those with filamentous lesions were not (*p* < .001, both valves).

**Conclusions:**

In a large academic center, the inter‐observer agreement for the presence of a left sided valvular mass was moderate and agreement regarding the final diagnosis of IE was fair to moderate, with better agreement among readers evaluating the MV. Lesion morphology is associated with the clinical diagnosis of IE.

## 1 INTRODUCTION

Despite advances in therapy, the incidence of infective endocarditis in the United States has continued to increase and morbidity and mortality remain high.^1^, ^2^ Echocardiography plays a vital role in the diagnosis and management of infective endocarditis (IE). Evidence of endocardial involvement on echocardiogram is a major criterion in the modified Duke criteria and the findings provide prognostic information to guide management.^3^, ^4^ Transesophageal echocardiography (TEE) is the gold standard for the detection of valvular vegetations with a high sensitivity and specificity of over 90 percent.^5^, ^6^ However, specificity depends on differentiating a valvular vegetation from other intracardiac masses, degenerative changes, and artifacts. Unfortunately, data on specificity is more limited because most studies comparing TEE to pathological findings include only subjects with definite endocarditis and subjects that are either sick enough to undergo surgery or who have died.^6^ In addition, what constitutes a vegetation is somewhat vague. It is typically described as an abnormal echogenic, irregular mass usually attached to a valve with independent motion.^6^, ^7^ Although, there are some features that can raise suspicion for IE, differentiating a small vegetation from other chronic valvular abnormalities is challenging and subject to reader interpretation. Advances in TEE technology and image quality have allowed for improved detection of vegetations but may also potentially lead to more false positive findings by detecting previously unseen small cardiac masses or chronic degenerative material attached to heart valves.

To our knowledge, there is limited data evaluating inter‐observer variability for valvular findings on TEEs performed for suspected IE. Our study aims to evaluate the level of inter‐observer agreement regarding aortic (AV) and mitral valve (MV) findings on TEEs ordered for suspected IE or bacteremia at a large, academic center. We also evaluated whether certain valvular mass characteristics are more likely to be associated with a final interpretation of IE.

## 2 MATERIALS AND METHODS

### 2.1 Study population

**FIGURE 1:**
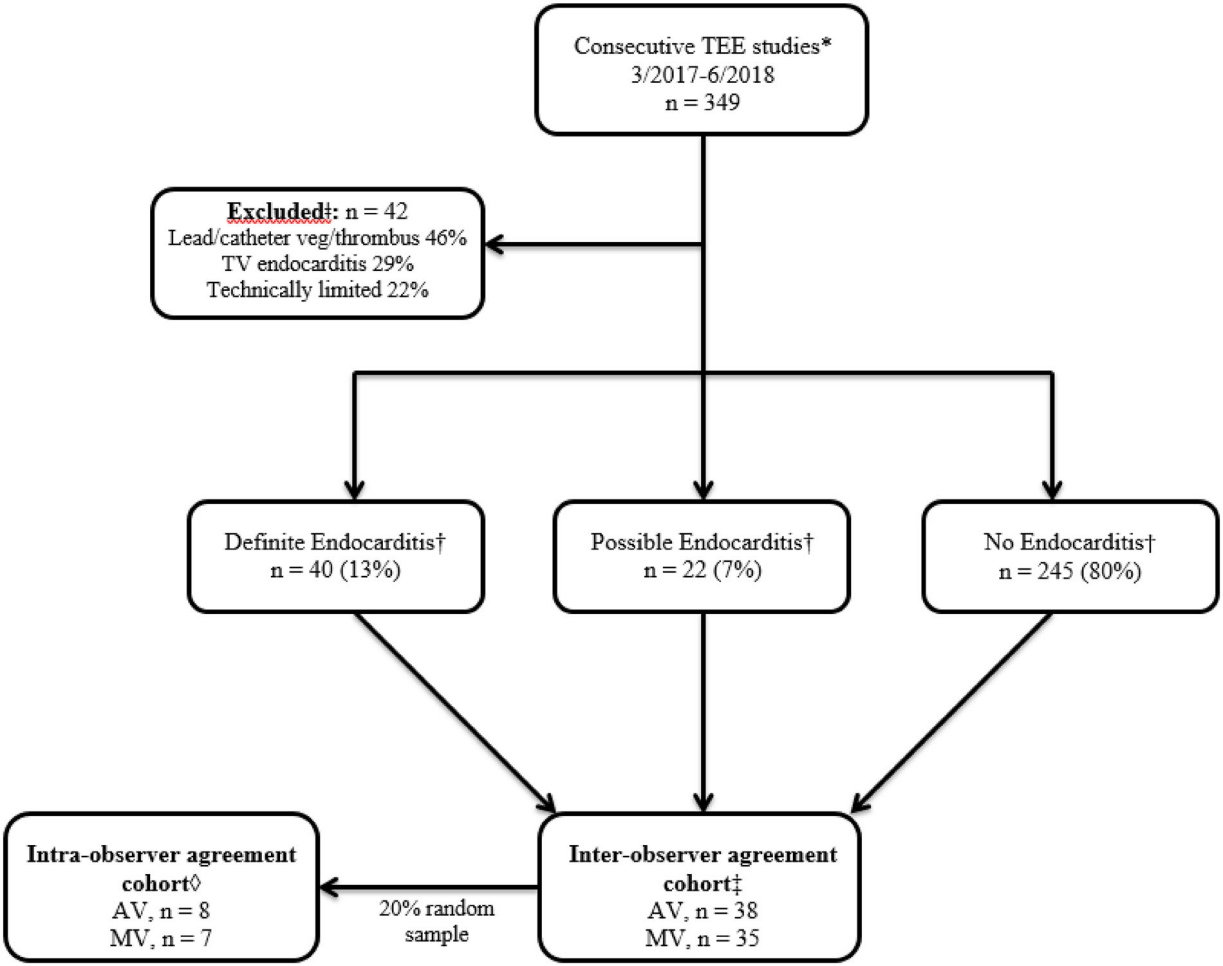
Consort diagram. *There were 349 consecutive TEE studies performed for suspected IE or bacteremia. ǂStudies that demonstrated right sided IE, catheter/device associated IE or thrombus, or were technically limited were excluded. †The remaining studies (*n* = 307) were classified as “definite, possible, or no” endocarditis based on the original TEE report interpretation. ‡A random sample of studies from each category were chosen for inter‐observer agreement for a total of 38 AV and 35 MV studies.

We conducted a retrospective review of 349 consecutive TEEs done between March 2017 and June 2018 in adults aged 18 years and older ordered for the clinical indications of bacteremia and/or suspected endocarditis in the adult echocardiography laboratory at the University of Wisconsin Hospital. The TEEs were performed using the Siemens SC2000 (Mountain View, CA) with a Siemens 3‐DTEE probe or the Philips EpiQ 7 with either a 7XT TEE probe or an 8XT TEE probe (Andover, MA) ultrasound imaging systems equipped with standard TEE transducers. Comprehensive studies were performed using both 2D and 3D interrogation. As our primary interest was assessing interpretation and level of agreement over what constitutes a vegetation versus degenerative change, we chose to focus on the left sided valves. Similarly, we wanted to assess agreement when valves were adequately visualized as agreement is expected to be lower when valves are not well visualized as demonstrated by Connolly et al.^8^ Therefore, we excluded studies that were reported as technically limited or demonstrated tricuspid, pulmonic, or catheter/device associated IE or thrombus (*n* = 42). After applying the exclusion criteria, three echocardiographers (K.M., M.T., and P.R.) classified the remaining 307 studies as “definite,” “possible,” or “no” infective endocarditis based on the original TEE report (definite, *n* = 40; possible, *n* = 22; no, *n* = 245). The “definite” and “possible” endocarditis studies were classified as an AV or MV study based on the valve involved. To minimize expectancy bias, a random sample of studies from each category was chosen with a total of 38 AV and 35 MV studies. See Figure 1.

### 2.2 Inter‐observer agreement

Three echocardiographers (K.M., M.T., and P.R.) reviewed each study and extracted 5–10 video loops of the AV or MV that were a full representation of the valvular findings and included different transducer angles, color Doppler, and 3D images when available. Nine level III faculty echo readers were recruited to review the de‐identified studies and completed a questionnaire for each valve (Tables S1 and S2) regarding valvular findings and specifically whether a valvular mass was present, the characteristics of the mass, and if the findings were consistent with endocarditis or not. Inter‐observer agreement was calculated among the nine readers based on the answers provided in the questionnaire and not in comparison to the original TEE report. The readers were aware that the study indication was bacteremia/suspected IE, otherwise were blinded to all other clinical or identification information.

### 2.3 Valvular mass characteristics and interpretation

Each of the 38 AV and 35 MV studies were assessed by nine readers for a total of 342 AV and 315 MV reader assessments. We evaluated the number of times an AV and MV mass were described out of the total number of reader assessments completed for inter‐observer agreement. For the assessments that noted a valvular mass, we compared individual mass characteristics to the final diagnosis of IE to see whether certain characteristics were more frequently associated with IE.

### 2.4 Clinical assessment of infective endocarditis

We conducted a retrospective chart review of the selected AV and MV studies to evaluate the likelihood of IE by assessing the components of the modified Duke criteria. The original TEE interpretation was used to calculate the modified Duke criteria. We also reviewed the final clinical diagnosis and management, including use and duration of antibiotics and valve surgery.

### 2.5 Statistical analyses

Descriptive statistics are reported as means (standard deviations [SD]) for numeric data and percentages/frequencies for categorical variables. Group comparisons were conducted using a student's *t*‐test for continuous variables. For categorical data such as patient demographics, valve lesion morphology assessment, and final diagnosis distributions, we used a chi‐squared test. All comparisons in which any cells had less than five observations was analyzed using a Fisher's exact test instead of the chi‐squared test. Interrater agreement was assessed using the KAPPAETC package with listwise correction of missing ratings and bootstrap for confidence intervals.^9^, ^10^ We used Fleiss/Scott's Kappa (K*_f_*) to estimate the corrected agreement for precision.^11^ All *p*‐values less than or equal to .05 were considered to be statistically significant. Analyses were performed in SAS (Version 9.2, Cary, NC: SAS. Institute Inc.) and STATA SE 16 (StataCorp, 2019. Stata Statistical Software: Release 19. College Station, TX).

## 3 RESULTS

### 3.1 Descriptive characteristics

The mean (SD) age of the participants overall at the time of the study was 60.1 (16.8) years with 70% male (Table 1). Baseline clinical factor distributions did not significantly vary between the AV and MV cases, except, there was a slightly higher rate of congenital heart disease in the AV studies (21.1% vs. 2.9%), *p* = .03, with the majority related to bicuspid AV (63%). Overall, the majority of participants (80.8%) had positive blood cultures with the most common organisms being staph aureus (42.5%) and viridans group streptococci (20.5%). Approximately, one third had known valvular disease (35.6%). A total of 14 patients (19.2%) had history of a prosthetic valve replacement, although only 10 of these prosthetic valves were evaluated (AV, *n* = 8; MV, *n* = 2). By modified Duke criteria, 45.2% had definite and 39.7% had possible IE. Final clinical diagnosis was definite IE in 46% and possible in 14%. The majority (89%) received antibiotics with average duration of 6.2 weeks in patients with definite or possible IE and 5.2 weeks in patients without IE. Only seven patients underwent valve surgery (9.6% of total patients, 16% of patients with definite, or possible IE). There were eight patients that died during the admission or were discharged to hospice (six had definite and one possible IE).

**TABLE 1:** Patient characteristics

	**Total *N* = 73**	**MV Studies *N* = 35**	**AV Studies*N* = 38**	***p*‐value**
Age (years), Mean + SD	60.1 (16.8)	59.1 (16.3)	61.0 (17.5)	.63^a^
Gender				
Male	51 (69.9)	24 (68.6)	27 (71.1)	.82^b^
Female	22 (30.1)	11 (31.4)	11 (28.9)	
Known Valve Disease, *n* (%)				
Yes	26 (35.6)	11 (31.4)	15 (39.5)	.47^b^
No	47 (64.4)	24 (68.6)	23 (60.5)	
Prosthetic Valve, *n* (%)				
Yes	14 (19.2)	5 (14.3)	9 (23.7)	.38^c^
No	59 (80.8)	30 (85.7)	29 (76.3)	
Type of Prosthetic Valve, *n* (%)				
Bio	11 (78.6)	4 (80)	7 (77.8)	1.0^c^
Mechanical	3 (21.4)	1 (20)	2 (22.2)	
Location of prosthetic Valve, *n* (%)				
AV	11 (78.6)	3 (60)	8 (88.9)	.51^c^
MV	3 (21.4)	2 (40)	1 (11.1)	
Congenital Heart Disease, *n* (%)				
Yes	9 (12.3)	1 (2.9)	8 (21.1)	**.03** **^c^** *
No	64 (87.7)	34 (97.1)	30 (78.9)	
History of Endocarditis, *n* (%)				
Yes	2 (2.7)	1 (2.9)	1 (2.6)	1.0^c^
No	71 (97.3)	34 (97.1)	37 (97.4)	
IV Drug Use, *n* (%)				
Yes	9 (12.3)	5 (14.3)	4 (10.5)	.73^c^
No	64 (87.7)	30 (85.7)	34 (89.5)	
Blood Culture result, *n* (%)				
Negative	14 (19.2)	5 (14.3)	9 (23.7)	.53^c^
MRSA	20 (27.4)	9 (25.7)	11 (28.9)	
MSSA	11 (15.1)	5 (14.3)	6 (15.8)	
Viridians Strep Group	15 (20.6)	9 (25.7)	6 (15.8)	
Coagulase Negative Staph	3 (4.1)	1 (2.9)	2 (5.3)	
Enterococcus	6 (8.2)	2 (5.7)	4 (10.5)	
Gram Negative Bacteremia	2 (2.7)	2 (5.7)	0 (0)	
Atypical Strep	2 (2.7)	2 (5.7)	0 (0)	
Fever, *n* (%)				
Yes	50 (68.5)	26 (74.3)	24 (63.2)	.31^b^
No	23 (31.5)	9 (25.7)	14 (36.8)	
Evidence of Emboli, *n* (%)				
Yes	29 (39.7)	15 (42.9)	14 (36.8)	.60^b^
No	44 (60.3)	20 (57.1)	24 (63.2)	
Immune Complex, *n* (%)				
Yes	3 (4.1)	1 (2.9)	2 (5.3)	1.0^c^
No	70 (95.9)	34 (97.1)	36 (94.7)	
Infective Endocarditis by Modified Duke Criteria, *n* (%)				
No	11 (15.1)	4 (11.4)	7 (18.4)	.77^c^
Definite	33 (45.2)	17 (48.6)	16 (42.1)	
Possible	29 (39.7)	14 (40)	15 (39.5)	
Endocarditis on Initial TEE Report, *n* (%)				
No	20 (27.4)	10 (28.6)	10 (26.2)	.86^b^
Maybe	21 (28.8)	9 (25.7)	12 (31.6)	
Yes	32 (43.8)	16 (45.7)	16 (45.1)	
Antibiotic Therapy, *n* (%)				
Yes	65 (89.0)	31 (88.6)	34 (89.5)	1.0^c^
No	8 (11.0)	4 (11.4)	4 (10.5)	
Valve Surgery, *n* (%)				
Yes	7 (9.6)	2 (5.7)	5 (13.2)	.43^c^
No	66 (90.4)	33 (94.3)	33 (86.8)	

^a^ Student t‐test.

^b^ Chi‐squared test.

^c^ Fisher's exact.

*Statistically significant at *p* ≤ .05.

### 3.2 Inter‐observer agreement

**FIGURE 2:**
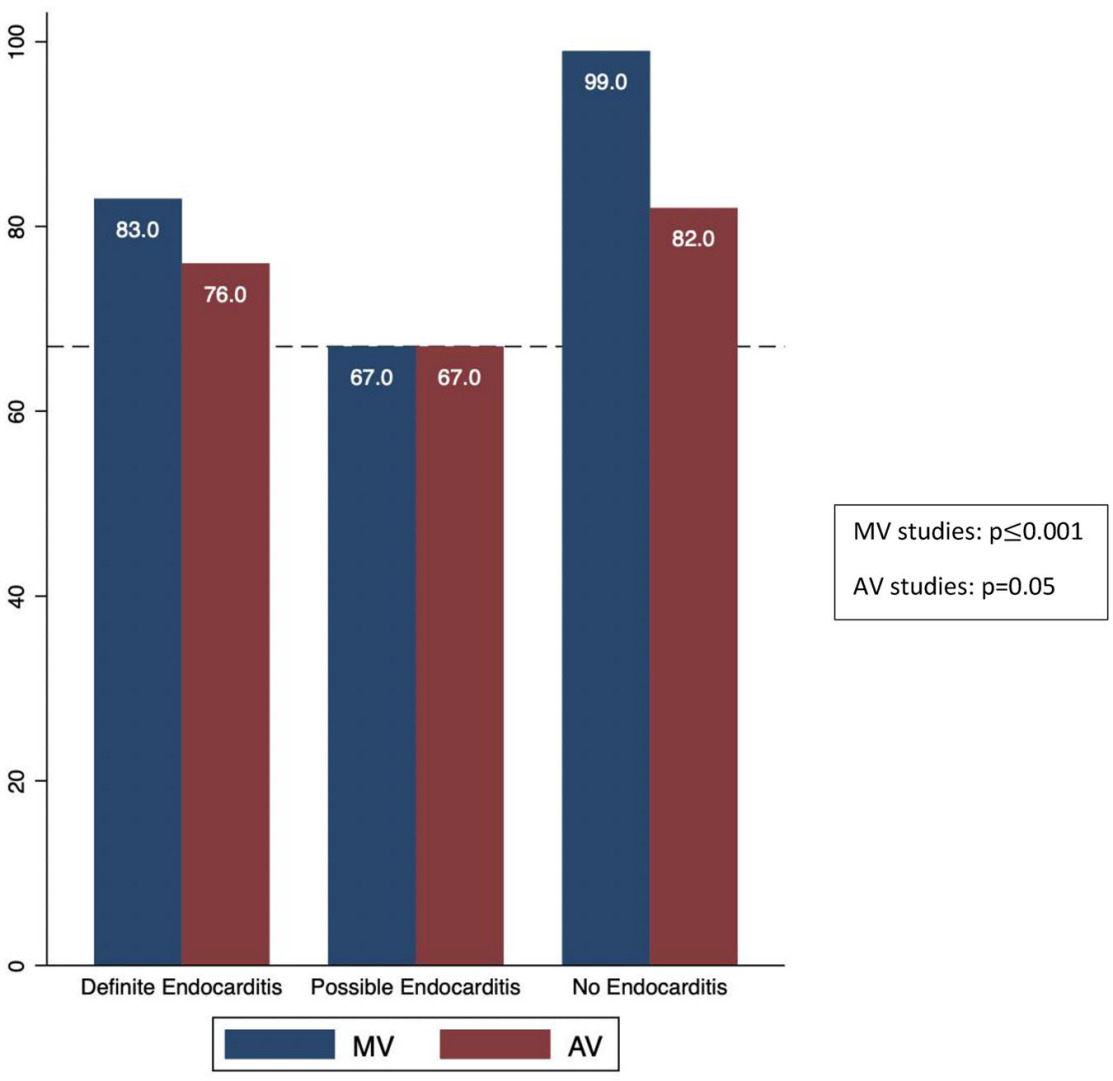
Inter‐observer Agreement Compared to Original TEE Interpretation. Studies were classified as “definite,” “possible,” or “no” endocarditis based on the original TEE report interpretation. Inter‐observer agreement was highest among studies classified as “no endocarditis” and the lowest among studies classified as “possible endocarditis.”

Regarding the presence of a valvular mass, inter‐observer agreement was 67% for the AV (*Kf* = .41, 95% CI [.30–.53]) and 72% for the MV (*Kf* = .49, 95% CI [.34–.65]). For the question, “are findings suggestive of endocarditis,” inter‐observer agreement was 66% for the AV (*Kf *= .29, 95% CI [.18–.42]) and 77% for the MV (*Kf *= .53, 95% CI [.36–.70]). For the assessment of individual morphologic characteristics traditionally felt to be consistent with a vegetation, the level of agreement was also moderate. The valve lesion characteristics are summarized in Table 2. The percent agreement for each individual study regarding the final interpretation of IE ranged from 56% to 100% for both the AV and MV (Table 3). Inter‐reader agreement was highest among studies that were interpreted as negative for IE on the original TEE report (AV = 82%, MV = 99%) and lowest for studies originally interpreted as possible IE (AV = 67%, MV = 67%) (AV, *p* = .05; MV, *p* ≤ .001) (Figure 2).

**TABLE 2:** Inter‐reader agreement of aortic and mitral valve findings on TEE

	**Aortic Valve (*n* = 38)**				**Mitral Valve (*n* = 35)**			
	# Of rating categories	Percent agreement (%)	K_f_	95% CI (K_f_)	# Of rating categories	Percent agreement (%)	K_f_	95% CI (K_f_)
Presence of valvular mass^a^	3	67	.41	.30–.53	3	72	.49	.34–.65
Side of the valve^b^	3	57	.38	.30–.48	3	71	.50	.35–.65
Cusp(s) or leaflets involved^c^	4	48	.30	.21–.39	3	68	.51	.37–.66
Motion of the mass^d^	2	67	.43	.30–.57	2	67	.47	.34–.60
Filamentous or Strand‐like^e^	2	64	.45	.34–.57	2	77	.63	.50–.76
Protruding or pedunculated^e^	2	56	.34	.25–.43	2	68	.51	.40–.63
Multi‐lobulated or irregularly shaped^e^	2	57	.34	.24–.44	2	67	.50	.37–.64
Estimated size^f^	3	58	.42	.33–.51	3	66	.53	.41–.66
Perivalvular regurgitation^e^	2	95	.55	.26–.85	2	97	.70	.31–1.0
Abscess^e^	2	92	.21	.05–.38	2	93	.23	.05–.40
Prosthetic valve dehiscence^g^	3	83	.54	.39–.70	3	93	.59	.26–.92
Leaflet damage^h^	3	75	.31	.16–.45	3	77	.28	.14–.41
Are findings suggestive of endocarditis?^e^	2	66	.29	.18–.42	2	77	.53	.36–.69
If no, what are the findings most consistent with?^i^	5	43	.23	.16–.31	4	65	.47	.36–.58

Abbreviation: K_f_, Scott/Fleiss kappa; 95% CI, 95% Confidence Intervals.

^a^ Yes, no, or possible.

^b^ Upstream, downstream, or both.

^c^ Right coronary cusp, left coronary cusp, non‐coronary cusp, unable to determine for AV; anterior, posterior or both for MV.

^d^ Mobile or sessile.

^e^ Yes or no.

^f^ Small, medium, or large.

^g^ Yes, no, or not applicable.

^h^ Intact, leaflet damage, leaflet perforation.

^i^ Benign stranding, thrombus, papillary fibroma, normal, Lambl's excresence.

**TABLE 3:** Individual study percent agreement regarding final interpretation of infective endocarditis

**Aortic Valve**		**Mitral Valve**	
AV Study #	Percent Agreement (%)	MV Study #	Percent Agreement (%)
1	100	1	67
2	78	2	100
3	89	3	89
4	89	4	56
5	56	5	100
6	67	6	89
7	56	7	78
8	56	8	67
9	67	9	100
10	89	10	78
11	67	11	100
12	89	12	56
13	89	13	56
14	89	14	100
15	56	15	89
16	89	16	78
17	56	17	56
18	67	18	100
19	100	19	100
20	100	20	100
21	78	21	100
22	89	22	100
23	100	23	56
24	67	24	56
25	56	25	67
26	78	26	67
27	67	27	100
28	78	28	100
29	89	29	100
30	56	30	100
31	89	31	67
32	100	32	67
33	89	33	100
34	56	34	100
35	89	35	100
36	56		
37	89		
38	67		

### 3.3 Valvular mass characteristics and interpretation

**FIGURE 3:**
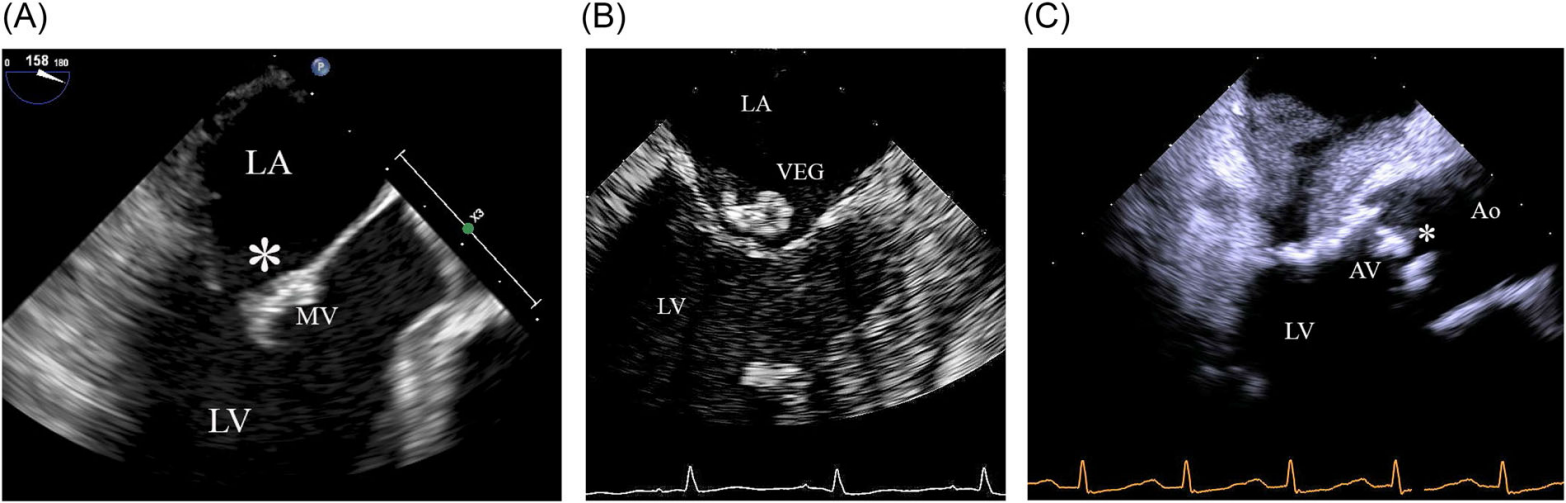
Examples of three different types of masses identified in the study. In (A) there is thickening of the anterior leaflet and prolapse (*). The vote was 5–4. Please also see Video 3A. In (B) there is a mobile mass on the atrial side of the mitral leaflet (*). Please see Video 3B. The vote was 9–0. In (C) the focus is on the AV that shows reduced motion, nodular calcium, and small mobile lesions attached to both visible leaflets (*). Please also see Video 3B. The vote 5–4. Ao = aorta LA = Left atrium, LV = left ventricle, MV = mitral valve, VEG = vegetation

An AV mass was noted in 62% of all reader assessments (*n* = 212) and 64% of these masses were interpreted as IE (*n* = 136). A MV mass was noted in 61% of all assessments (*n* = 193) with 72% of these masses being interpreted as IE (*n* = 139). Examples of masses evaluated are shown in Figure 3A–C and corresponding Videos 3A, 3B, and 3C. Valvular mass characteristics that were associated more frequently with a final interpretation of IE for both the AV and MV included large size, multi‐lobulated and/or irregularly shaped, and protruding and/or pedunculated (*p* ≤ .006 for both valves). Patients diagnosed with infective endocarditis were less likely to have a filamentous mass (AV = 33.3%, MV = 20.4%) compared to those with no endocarditis (AV = 66.2%, MV = 54.7% *p* < .001 for both) (Figure 3). For medium sized masses, the shape of the mass did not make a significant difference in interpretation, except for masses described as multi‐lobulated and/or irregularly shaped which were more frequently interpreted as IE (AV, *p* = .01; MV, *p* = .002) Table 5. There was no difference in interpretation based on the motion of the mass (AV, *p* = .84; MV, *p* = .66). See Table 4.

**VIDEO 3A:** Video 3A corresponds to figure 3A. There is thickening of the anterior leaflet of the mitral valve.

**VIDEO 3B:** Video 3B corresponds to figure 3B. Note the atrial mass on the atrial side of the mitral valve.

**VIDEO 3C:** Video 3C corresponds to figure 3C. There are calcified lesions on the aortic valve leaflets with small mobile lesions attached to each visible leaflet.

**TABLE 4:** Valvular mass characteristics and final interpretation

	**Aortic Valve Masses**				**Mitral Valve Masses**			
	Total*N* = 212	No Endocarditis*N* = 76	Endocarditis*N* = 136	*p*‐value	Total*N* = 193	No Endocarditis*N* = 54	Endocarditis*N* = 139	*p*‐value
Which side of the valve is it located?								
Upstream	112 (53.6)	42 (56.8)	70 (51.8)	<.001*^,^ **^a^**	162 (84.8)	48 (90.6)	114 (82.6)	.13^b^
Downstream	51 (24.4)	27 (36.5)	24 (17.8)		8 (4.2)	3 (5.7)	5 (3.6)	
Both	46 (22.0)	5 (6.8)	41 (30.4)		21 (11)	2 (3.8)	19 (13.8)	
What is the motion of the mass?								
Mobile	166 (80.2)	58 (79.4)	108 (80.6)	.84**^a^**	137 (71.7)	37 (69.8)	100 (72.5)	.71**^a^**
Sessile	41 (19.8)	15 (20.5)	26 (19.4)		54 (28.3)	16 (30.2)	38 (27.5)	
Is the mass filamentous or strand‐like?								
Yes	94 (45)	49 (66.2)	45 (33.3)	<.001*^,^ **^a^**	57 (30)	29 (54.7)	28 (20.4)	<.001*^,^ **^a^**
No	115 (55)	25 (33.8)	90 (66.7)		133 (70)	24 (45.3)	109 (79.6)	
Is the mass protruding and/or pedunculated?								
Yes	83 (39.7)	20 (27)	63 (46.7)	.006*^,^ **^a^**	109 (57.4)	21 (39.6)	88 (64.2)	.002*^,^ **^a^**
No	126 (60.3)	54 (73)	72 (53.3)		81 (42.6)	32 (60.4)	49 (35.8)	
Is the mass multi‐lobulated and/or irregularly shaped?								
Yes	76 (36.4)	9 (12.2)	67 (49.6)	<.001*^,^ **^a^**	87 (45.8)	8 (15.1)	79 (57.7)	<.001*^,^ **^a^**
No	133 (63.6)	65 (87.8)	68 (50.4)		103 (54.2)	45 (84.9)	58 (42.3)	
Estimated size of largest vegetation or mass?								
Small (0–5 mm)	92 (44.2)	50 (68.5)	42 (31.1)	<.001*^,^ **^a^**	76 (40)	34 (64.1)	42 (30.7)	<.001*^,^ **^b^**
Medium (5–10 mm)	53 (25.5)	16 (21.9)	37 (27.4)		47 (24.7)	15 (28.3)	32 (23.4)	
Large (> 10 mm)	63 (30.3)	7 (9.6)	56 (41.5)		67 (35.3)	4 (7.6)	63 (46)	

^a^ Chi‐squared test.

^b^ Fisher's exact.

*Statistically significant at *p*≤.05;

**TABLE 5:** Valvular mass characteristics and final interpretation by readers for medium sized masses

	**Aortic Valve Masses, Medium mass only (**5–10 mm)				**Mitral Valve Masses Medium mass only (**5–10 mm)			
	Total *N* = 53	No Endocarditis *N* = 16	Endocarditis *N* = 37	*p*‐value	Total *N* = 47	No Endocarditis *N* = 15	Endocarditis *N* = 32	*p*‐value
Is the mass filamentous or strand‐like?								
Yes	22 (41.5)	9 (56.2)	13 (35.1)	.15	18 (38.3)	8 (53.3)	10 (31.2)	.15
No	31 (58.5)	7 (43.8)	24 (64.9)		29 (61.7)	7 (46.7)	22 (68.8)	
Is the mass protruding and/or pedunculated?								
Yes	28 (52.8)	7 (43.8)	21 (56.8)	.38	28 (59.6)	6 (40.0)	22 (68.8)	.06
No	25 (47.2)	9 (56.2)	16 (43.2)		19 (40.4)	9 (60.0)	10 (31.2)	
Is the mass multi‐lobulated and/or irregularly shaped?								
Yes	24 (45.3)	3 (18.8)	21 (56.8)	.01*	18 (38.3)	1 (6.7)	17 (53.1)	.002*
No	29 (54.7)	13 (81.2)	16 (43.2)		29 (61.7)	14 (93.3)	15 (46.9)	

*Statistically significant at *p *≤ .05; Fisher's exact.

## 4 DISCUSSION

In a single center evaluation of consecutive TEE studies performed for bacteremia/suspected IE, there was moderate inter‐observer agreement regarding the presence of a valvular mass and fair to moderate agreement regarding the diagnosis of IE, with a higher agreement among readers evaluating the MV.

The current study is distinctive in that it evaluates contemporary inter‐observer variability for left sided valve findings on TEEs performed for suspected IE among echocardiographers at an academic institution.

There are a limited number of published studies evaluating inter‐observer agreement for findings of IE by echocardiography and the majority of the subjects included in them had definite IE. Heinle et al. evaluated inter‐observer variability among four echocardiographers at a single center for the presence and characteristics of vegetations on transthoracic echocardiography (TTE) in 41 patients with IE.^12^ In this study, there was 98% agreement regarding vegetation presence (*K* = .73), but significant heterogeneity in agreement metrics (*K* = .39–.84) regarding vegetation characteristics and location. In another study by Kiilerich Lauridsen et al. inter‐ and intra‐observer agreement for IE echocardiography variables were assessed by comparing site interpretations to readings at a central echocardiography core laboratory for a random sample of 110 echocardiograms from the International Collaboration of Infective Endocarditis (ICE‐PCS) and intra‐observer measures were performed on a smaller sample from six site readers on 10 echocardiograms.^13^, ^14^ In this study, there was moderate to excellent intra‐ and inter‐observer agreement for echocardiographic variables in the evaluation of IE. Of note, this study included both TTE and TEE studies and the majority of studies had definite IE (85%) by modified Duke criteria. Compared to the studies conducted by Heinle et al. and Kiilerich Lauridsen et al., our study demonstrated significantly lower inter‐observer agreement regarding the presence of a valvular mass. This may be partially attributed to the design of our study. First, our study by design had a lower prevalence of “definite endocarditis” based on modified Duke criteria (45% vs. 85%) and higher prevalence of “possible” or “no” endocarditis. This difference in prevalence may have led to increased heterogeneity among readers due to different thresholds for what constitutes a valvular mass. Second, we required the reader to give a definitive answer regarding whether the findings were consistent with IE, whereas the other studies did not. Additionally, the current study only included TEE studies compared to only TTEs with Heinle et al. and both TEEs and TTEs with Kiilerich Lauridsen et al. TEE has a higher resolution and has the potential to detect smaller valvular abnormalities that are subject to reader interpretation. Although, these factors likely contributed to lower inter‐observer agreement, it reflects a more real‐world setting.

There was significant variability in inter‐observer agreement between the studies over whether the findings were suggestive of IE. Inter‐observer agreement over the final interpretation was higher among the MV studies with 100% agreement seen in 17 studies compared to only six studies for the AV (Table 3). Inter‐observer agreement was as low as 56% in nine AV and six MV studies, indicating that there can be significant variation in how valvular findings are interpreted. Not surprisingly, agreement among readers was lowest for studies that were interpreted as “possible endocarditis” on the original TEE report, which further demonstrates the challenge of differentiating a vegetation from other valvular abnormalities. Inter‐observer agreement could potentially be improved by holding regular quality assurance reviews to discuss more challenging cases and with better recognition of degenerative related valvular changes.

To our knowledge, this is the first study looking at individual valvular mass characteristics that might be associated with reader interpretation of IE. Not surprisingly, masses that were larger in size, multi‐lobulated, or protruding were more frequently interpreted as consistent with IE. Multi‐lobulated shape also seemed to be the most indicative characteristic of IE in medium sized masses. The mobility of the mass, a frequently cited characteristic of vegetations, did not have an association with the final interpretation for IE. This finding was unexpected given a key characteristic of VV is independent motion, but oftentimes degenerative changes such as benign stranding/Lambl's are mobile as well. In addition, location of the mass did not differentiate IE from degenerative changes. Only those subjects with a mass on both sides of the valve had a significant association with IE. As many of the characteristic features of vegetations are also commonly seen with degenerative changes, this highlights the challenge of interpretating findings that are not obvious. In such cases, evidence of valvular dysfunction, such as regurgitation and leaflet damage, can be clues to IE. Clinical information should also be utilized whenever available to help with interpretation, such as reviewing prior echocardiograms to assess whether findings are new or chronic and evaluating the other components of the modified Duke criteria to estimate the likelihood of IE. Further studies are needed to determine which valvular mass characteristics may be most indicative of IE. There is also a significant need to characterize the nature of degenerative changes in high resolution TEE studies in patients with no suspicion of infection.

### 4.1 Limitations

This study has several limitations. First, the gold standard diagnosis of valvular endocarditis is based on histological analysis of surgical or autopsy data and very few patients in the study underwent surgery. In addition, the clinical diagnosis and calculated modified Duke criteria were based off the original TEE report, therefore the accuracy of the observer interpretations is difficult to determine. However, the purpose of our investigation was not to define the accuracy of an echocardiographer's reads, but rather to investigate the agreement of valvular mass identification and characterization among experienced echocardiographers. Second, the readers had no clinical information aside from the study indication. Knowledge of additional clinical data and patient risk factors can have a significant influence (bias) on how valvular findings are interpreted, therefore the lack of this additional information may have affected the readers’ answers. However, the indication for the study was “positive blood cultures” which ensured the reader would be evaluating the valve structures for infectious sequelae. Third, our study was performed at a single academic center in the United States and the generalizability to other centers or settings may be limited.

## 5 CONCLUSION

This is a single center study of TEEs performed for bacteremia/suspected IE, the inter‐observer agreement for the presence of a left sided valvular mass was moderate and agreement regarding the final diagnosis of IE was fair to moderate, with a higher agreement among readers evaluating the MV. Masses that were described as large, multi‐lobulated, or protruding/pedunculated and on both sides of the valve leaflet were more frequently associated with a final interpretation of IE. This study highlights contemporary echocardiographer interpretation agreement for left sided valvular endocarditis. In an era of improved resolution of TEE images, these data support the need for further investigations to improve the evaluation of structural changes seen on cardiac valves. Recognition of minor changes in structure rarely associated with IE may reduce the length and intensity of antibiotic treatment.

## CONFLICT OF INTEREST

None declared.

## Supporting information

Supporting Information.
