# Supplementary material for: Evaluation of inter‐observer variability regarding aortic and mitral valve findings on transesophageal echocardiograms ordered for suspected endocarditis
Source: Echocardiography. 2022 Jun 22;39(7):906–17. doi: 10.1111/echo.15400 (PMC9541542; doi:10.1111/echo.15400)
Supplement: Supplementary file 1 — Supporting Information. [file ECHO-39-906-s001.docx]

**Table 1: Transesophageal Echocardiogram Aortic Valve Questions:**

Study number: _________

1. Image Quality:
   1. Poor
   2. Fair
   3. Good
2. Valve status?
   1. Native
   2. Bicuspid
   3. Bioprosthetic
   4. Mechanical
3. Regurgitation?
   1. None or trivial
   2. Mild
   3. Moderate
   4. Severe
4. Perivalvular regurgitation?
   1. Yes
   2. No
5. Abscess?
   1. Yes
   2. No
6. Prosthetic valve dehiscence?
   1. Yes
   2. No
   3. N/A
7. Presence of other valvular abnormalities? List all that apply.
   1. Calcification
   2. Thickening
8. Valve integrity?
   1. Functional/intact
   2. Evidence of leaflet damage
   3. Evidence of leaflet perforation
9. Presence of valvular mass?
   1. No
   2. Yes
   3. Possible

If answer to question 9 is yes or possible then answer questions 10-16**. If no, skip to question 17.**

1. Which side of the valve is it located?
   1. Aortic
   2. Ventricular
   3. Both
2. Which cusp(s)? Circle all that apply
   1. Right coraonary cusp
   2. Left coronary cusp
   3. Non coronary cusp
   4. Unable to determine
3. What is the motion of the mass?
   1. Mobile
   2. Sessile
4. Is the mass filamentous or strand-like?
   1. Yes
   2. No
5. Is the mass protruding and/or pedunculated?
   1. Yes
   2. No
6. Is the mass multi-lobulated and/or irregularly shaped?
   1. Yes
   2. No
7. Estimated size of largest vegetation or mass?
   1. Small (0-5 mm)
   2. Medium (5-10 mm)
   3. Large (>10 mm)
8. Are findings suggestive of aortic valve endocarditis?
   1. Yes
   2. No
9. If the answer to question 17 is no, what are the findings most consistent with?
   1. Benign stranding
   2. Thrombus
   3. Lambl’s excrescence
   4. Papillary fibroma
   5. Normal valve

**Table 2: Transesophageal Echocardiogram Mitral Valve Questions:**

Study number: _________

1. Image Quality:
   1. Poor
   2. Fair
   3. Good
2. Valve status:
   1. Native
   2. Native with ring
   3. Bioprosthetic
   4. Mechanical
3. Regurgitation?
   1. None or trivial
   2. Mild
   3. Moderate
   4. Severe
4. Perivalvular regurgitation?
   1. Yes
   2. No
5. Abscess?
   1. Yes
   2. No
6. Prosthetic valve dehiscence?
   1. Yes
   2. No
   3. N/A
7. Presence of other valvular abnormalities? List all that apply
   1. Calcification
   2. Thickening
   3. Prolapse
8. Valve integrity?
   1. Functional/intact
   2. Evidence of leaflet damage
   3. Evidence of leaflet perforation
9. Presence of valvular mass?
   1. No
   2. Yes
   3. Possible

If answer to question 9 is yes or possible then answer questions 10-16. **If no, skip to question 17.**

1. Which side of the valve is it located?
   1. Atrial
   2. Ventricular
   3. Both
2. Which leaflet(s)?
   1. Anterior
   2. Posterior
   3. Both
3. What is the motion of the mass?
   1. Mobile
   2. Sessile
4. Is the mass filamentous or strand-like?
   1. Yes
   2. No
5. Is the mass protruding and/or pedunculated?
   1. Yes
   2. No
6. Is the mass multi-lobulated and/or irregularly shaped?
   1. Yes
   2. No
7. Estimated size of largest vegetation or mass?
   1. Small (0-5 mm)
   2. Medium (5-10 mm)
   3. Large (>10 mm)
8. Are findings suggestive of mitral valve endocarditis?
   1. Yes
   2. No
9. If the answer to question 17 is no, what are the findings most consistent with?
   1. Benign stranding/myxomatous degeneration
   2. Thrombus
   3. Papillary fibroma
   4. Normal valve
